# Microdeletion in a *FAAH* pseudogene identified in a patient with high anandamide concentrations and pain insensitivity

**DOI:** 10.1016/j.bja.2019.02.019

**Published:** 2019-03-28

**Authors:** Abdella M. Habib, Andrei L. Okorokov, Matthew N. Hill, Jose T. Bras, Man-Cheung Lee, Shengnan Li, Samuel J. Gossage, Marie van Drimmelen, Maria Morena, Henry Houlden, Juan D. Ramirez, David L.H. Bennett, Devjit Srivastava, James J. Cox

**Affiliations:** 1 Molecular Nociception Group, Wolfson Institute for Biomedical Research, University College London, London, UK; 2 College of Medicine, Member of Qatar Health Cluster, Qatar University, Doha, Qatar; 3 Hotchkiss Brain Institute, Departments of Cell Biology and Anatomy and Psychiatry, University of Calgary, Calgary, AB, Canada; 4 UK Dementia Research Institute at UCL, London, UK; 5 Department of Molecular Neuroscience, Institute of Neurology, University College London, London, UK; 6 University Division of Anaesthesia, University of Cambridge, Addenbrooke's Hospital, Hills Road, Cambridge, UK; 7 Department of Anesthesia and Perioperative Care, University of California, San Francisco, San Francisco, CA, USA; 8 Department of Pathology, Raigmore Hospital, Inverness, UK; 9 Nuffield Department of Clinical Neurosciences, University of Oxford, Oxford, UK; 10 Department of Anaesthesia, Raigmore Hospital, Inverness, UK

**Keywords:** anandamide, anxiolytic, endocannabinoids, pain insensitivity, postoperative analgesia

## Abstract

The study of rare families with inherited pain insensitivity can identify new human-validated analgesic drug targets. Here, a 66-yr-old female presented with nil requirement for postoperative analgesia after a normally painful orthopaedic hand surgery (trapeziectomy). Further investigations revealed a lifelong history of painless injuries, such as frequent cuts and burns, which were observed to heal quickly. We report the causative mutations for this new pain insensitivity disorder: the co-inheritance of (i) a microdeletion in dorsal root ganglia and brain-expressed pseudogene, *FAAH-OUT*, which we cloned from the fatty-acid amide hydrolase (*FAAH*) chromosomal region; and (ii) a common functional single-nucleotide polymorphism in *FAAH* conferring reduced expression and activity. Circulating concentrations of anandamide and related fatty-acid amides (palmitoylethanolamide and oleoylethanolamine) that are all normally degraded by FAAH were significantly elevated in peripheral blood compared with normal control carriers of the hypomorphic single-nucleotide polymorphism. The genetic findings and elevated circulating fatty-acid amides are consistent with a phenotype resulting from enhanced endocannabinoid signalling and a loss of function of FAAH. Our results highlight previously unknown complexity at the *FAAH* genomic locus involving the expression of *FAAH-OUT*, a novel pseudogene and long non-coding RNA. These data suggest new routes to develop *FAAH*-based analgesia by targeting of *FAAH-OUT*, which could significantly improve the treatment of postoperative pain and potentially chronic pain and anxiety disorders.

Fatty-acid amide hydrolase (FAAH) is the major catabolic enzyme for a range of bioactive lipids called fatty-acid amides (FAAs).^1^, ^2^ These FAAs include *N*-acyl ethanolamines, such as anandamide (AEA), that act as endogenous ligands for cannabinoid receptors (i.e. endocannabinoids). Other substrates of FAAH include palmitoylethanolamide (PEA), oleoylethanolamine (OEA), and *N*-acyl-taurines. 2-Arachidonoylglycerol (2-AG) is another related endocannabinoid and FAA, but is metabolised mostly by monoacylglycerol lipase (MAGL). AEA has roles in nociception, fear-extinction memory, anxiety, and depression.^3^, ^4^ *FAAH* knockout mice have elevated brain concentrations of AEA, display an analgesic phenotype in response to acute thermal stimuli, and show reduced pain in formalin and carrageenan inflammatory models.^5^, ^6^ FAAH is therefore an attractive drug target for treating pain, anxiety, and depression, although recent clinical trials with FAAH inhibitors were unsuccessful.^7^, ^8^

The human *FAAH* gene contains a commonly carried hypomorphic single-nucleotide polymorphism (SNP) (C385A; rs324420; C allele frequency 74%, A 26%) that significantly reduces the activity of the FAAH enzyme.^9^ Genetic association studies have investigated the link between this and other *FAAH* SNPs and pain sensitivity.^10^, ^11^, ^12^ Notably, homozygous carriers of the hypomorphic SNP (A allele) in a cohort of women undergoing breast cancer surgery were less sensitive to cold pain and had a reduced need for postoperative analgesia.^10^ Furthermore, a mouse knock-in model of the human SNP showed that both the mouse and human SNP carriers display enhanced fear-extinction learning and decreased anxiety-linked behaviours.^13^ Here, we describe a pain-insensitive patient with a non-anxious disposition presenting with a novel genetic disorder associated with loss of function of *FAAH*.

## Case report

A 66-yr-old Caucasian female presented to Raigmore Hospital in Inverness, Scotland for orthopaedic surgery, specifically a trapeziectomy with ligament reconstruction and tendon interposition and *extensor pollicis longus* realignment after a diagnosis of bilateral pantrapezial osteoarthritis. There was significant deformity and deterioration in the use of the right thumb, which was reported as painless before operation. The pre-assessment note classed her as ASA physical status 1, but highlighted that she had a history of vomiting after intake of morphine.

For the surgery, she received general anaesthesia with an ultrasound-guided axillary nerve block. She received fentanyl 50 μg i.v., propofol 200 mg i.v., ondansetron 4 mg i.v. intraoperatively, and levobupivacaine 0.25% (20 ml) for the axillary nerve block. After operation, her pain intensity score was 0/10 until the next day when she was discharged home. The only postoperative analgesic she received in hospital was paracetamol 1 g i.v. in the PACU on the day of her surgery. She also received cyclizine 50 mg i.v. twice. Extraordinarily, she required no postoperative analgesics other than paracetamol for this known painful surgery (trapeziectomy), even after the axillary nerve block had worn off. She showed no pain from pinching or from peripheral i.v. cannula manipulation, which led to further investigations.

The patient had been diagnosed with osteoarthritis of the hip, which she reported as painless, which was not consistent with the severe degree of joint degeneration. At 65 yr of age, she had undergone a hip replacement and was administered only paracetamol 2 g orally on Postoperative days 1 and 2, reporting that she was encouraged to take the paracetamol, but that she did not ask for any analgesics. She was also administered a single dose of morphine sulphate 10 mg orally on the first postoperative evening that caused severe nausea and vomiting for 2 days. After operation, her pain intensity scores were 0/10 throughout except for one score of 1/10 on the first postoperative evening. Her past surgical history was notable for multiple varicose vein and dental procedures for which she has never required analgesia. She also reported a long history of painless injuries (e.g. suturing of a laceration and left wrist fracture) for which she did not use analgesics. She reported numerous burns and cuts without pain (Supplementary Fig. S1), often smelling her burning flesh before noticing any injury, and that these wounds healed quickly with little or no residual scar. She reported eating Scotch bonnet chilli peppers without any discomfort, but a short-lasting ‘pleasant glow’ in her mouth. She described sweating normally in warm conditions.

The patient lives with her husband, and has a daughter and a son from her previous marriage. Her family history is unremarkable for neuropathy or painful conditions. Her mother and daughter appear to perceive pain normally. Her father (now deceased) had little requirement for pain killers. Her son also reports of having some degree of pain insensitivity, but not to the same extent as her. She does not take any medication at present, and is fit and active with no medical conditions apart from arthritis (Supplementary Fig. S2). She is talkative and happy with an optimistic outlook. On the Generalized Anxiety Disorder-7 anxiety questionnaire taken at age 70, she scored 0/21, classified as mild (the lowest category).^14^ Likewise, on the Patient Health Questionnaire-9 for depression, she scored 0/29, classified as mild.^15^ She reported long-standing memory lapses (e.g. frequently forgetting words mid-sentence and placement of keys). She also reported never panicking, not even in dangerous or fearful situations, such as in a recent road traffic accident.

After the painless trapeziectomy surgery and a history of ‘painless operations’, she was referred to and further investigated by pain genetics teams from University College London and the University of Oxford at age 67 yr. Ethical approval was granted from both institutions, and written consent taken from the patient, her two children, and mother. On clinical examination, she had multiple scars around the arms and on the back of her hands. Quantitative sensory testing (Supplementary Fig. S3) demonstrated hyposensitivity to noxious heat both in the hands and feet (see Supplementary data for further clinical details).

### Genetic tests identify a microdeletion downstream of *FAAH*

**Fig 1:**
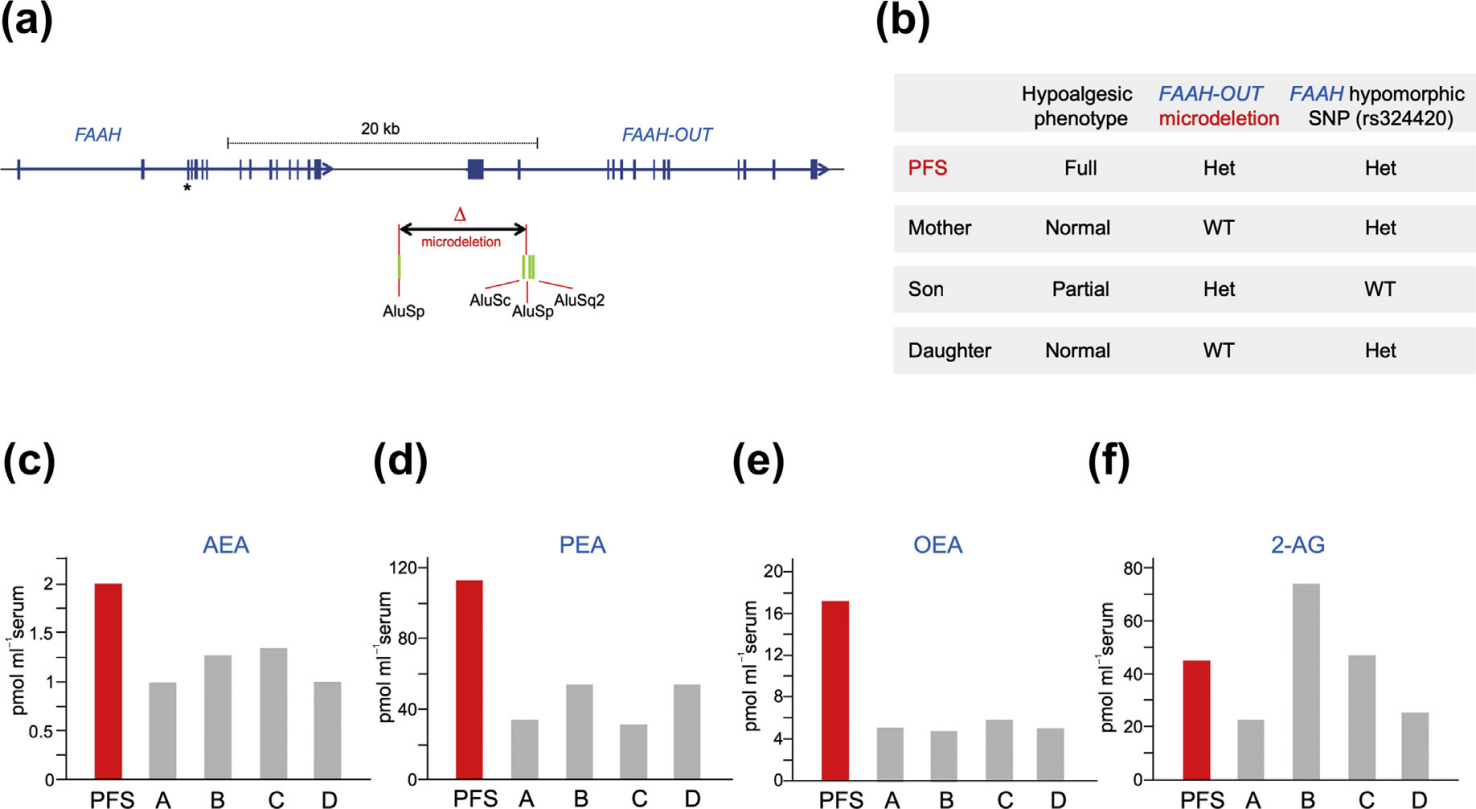
(a) Genomic map showing *FAAH*, *FAAH-OUT*, and microdeletion. Human chromosome 1 showing the gene footprints of *FAAH* and *FAAH-OUT*. Exons are denoted by blue boxes and the direction of transcription shown by arrows. *FAAH* single-nucleotide polymorphism (SNP) rs324420 maps to Exon 3 (indicated by an asterisk). The 8131 bp microdeletion detected in the patient is shown flanked by Alu repeated sequences (green boxes), which likely predispose the genomic region to rearrangements. The promoter region and Exons 1 and 2 of *FAAH-OUT* map to the deleted sequence. (b) Genotype summary. The proband carries both the *FAAH-OUT* microdeletion and the hypomorphic *FAAH* SNP, and displayed a full hypoalgesic phenotype. Her son carries the *FAAH-OUT* microdeletion and had a partial hypoalgesic phenotype. Neither the unaffected mother nor daughter carries the microdeletion. (c–f) Circulating anandamide (AEA), palmitoylethanolamide (PEA), oleoylethanolamine (OEA), and 2-arachidonoylglycerol (2-AG) concentrations. Concentrations of AEA, PEA, OEA, and 2-AG were measured by mass spectrometry from blood samples obtained from the patient and four unrelated normal controls. AEA, PEA, and OEA are substrates for FAAH; 2-AG is not. Controls A and B are homozygous wild type for the hypomorphic SNP; Controls C and D are heterozygous carriers. Average values for the controls were AEA (1.2 pmol ml^−1^), PEA (43.4 pmol ml^−1^), OEA (5.1 pmol ml^−1^), and 2-AG (42.2 pmol ml^−1^), which is consistent with previous data using a similar measurement protocol.^16^ Average values for the patient (two measurements) were AEA (2.0 pmol ml^−1^), PEA (113.1 pmol ml^−1^), OEA (17.3 pmol ml^−1^), and 2-AG (45 pmol ml^−1^).

Genomic DNA was isolated from the patient, her two children, and her mother for exome sequencing. After filtering of variants, four candidate mutations in the patient and her son were identified, but none were considered likely to be causal for the phenotype (see Supplementary data). We broadened our genetic analyses and searched for cytogenetic copy number changes across the genome using the CytoScan™ HD Array (Thermo Fisher Scientific, UK). This identified an ∼8 kb heterozygous microdeletion on Chromosome 1 that began ∼4.7 kb downstream from the 3′ end of *FAAH* (Fig 1a; Supplementary Fig. S4). Polymerase chain reaction and sequencing analyses confirmed that the patient co-inherited the microdeletion and *FAAH* hypomorphic SNP allele (rs324420) (Fig 1b). Her unaffected mother and daughter did not carry the microdeletion, but her son, who also has some pain-sensitivity deficits, was heterozygous for the microdeletion (Supplementary Fig. S5), but did not carry the hypomorphic SNP allele. One Colombian male (HG01353) (pain phenotype unknown) out of 5008 alleles screened in the 1000 Genomes Project also carries a similar-sized microdeletion (esv3585936 in Supplementary Table S1), but is homozygous wild type for *FAAH* SNP rs324420.

Given the extraordinary phenotype in the patient and the vicinity of the microdeletion to *FAAH*, we investigated how the microdeletion could be pathogenic. Molecular cloning experiments (see Supplementary data) identified novel 5′exons of an expressed *FAAH* pseudogene, herein called *FAAH-OUT* (2.845 kb cDNA; KU950306), that mapped to within the microdeletion (Fig 1a). Tissue expression analyses showed *FAAH-OUT* to be expressed in a wide range of tissues, including fetal and adult brain, and in dorsal root ganglia (DRG; Supplementary Fig. S6). *FAAH-OUT* likely encodes a long non-coding RNA (Supplementary Fig. S7). We considered that the microdeletion may negate the normal function of *FAAH* through a reduction in neural expression of *FAAH-OUT* or through loss of a critical genomic regulatory element for *FAAH*, and hence, obtained blood samples to measure *FAAH*-regulated lipids.

### Elevated FAA concentrations in blood

To determine the effects of carrying both the microdeletion in *FAAH-OUT* and the hypomorphic *FAAH* SNP, we measured the circulating FAA concentrations from blood samples from the patient and four controls, two of which were heterozygous carriers of the SNP. Circulating concentrations of AEA were increased by 70% and of OEA and PEA approximately tripled compared with controls (Fig. 1c–e). The concentrations of 2-AG, another endocannabinoid that is mainly degraded by MAGL and not FAAH, were largely unaltered (Fig 1f). These results are consistent with FAAH having a significant loss of function in the patient.

## Discussion

The endocannabinoid system is an important physiological system that performs a wide array of homeostatic functions and is important for pain perception.^17^ FAAH is a critical enzyme for the breakdown of a range of bioactive lipids (including the endocannabinoid AEA and related FAAs and *N*-acyl-taurines) with diverse physiological roles. Mouse modelling of FAAH loss of function mutations and pharmacological inhibition studies have shown a range of phenotypes, including hypoalgesia, accelerated skin wound healing, enhanced fear-extinction memory, reduced anxiety, and short-term memory deficits.^6^, ^13^, ^18^, ^19^, ^20^, ^21^ Furthermore, human hypomorphic *FAAH* SNPs are associated with a reduced need for postoperative analgesia, increased postoperative nausea and vomiting induced by opioids, and decreased anxiety-linked behaviours.^10^, ^13^, ^16^, ^22^, ^23^, ^24^

Here, we report a new human genetic disorder in a patient with hypoalgesia, altered fear and memory symptoms, and a non-anxious disposition. This disorder is attributable to co-inheritance of a microdeletion in a novel pseudogene and a known *FAAH* hypomorphic SNP. The microdeletion is flanked by repeated sequences that likely predispose the region to genomic rearrangements, as seen in other genomic disorders.^25^ Consequently, there are likely to be additional similar individuals in the general population. The likelihood that this disorder has been under-reported is highlighted by the fact that the patient was diagnosed at age 66 yr despite a recurrent history of painless injuries. Lipid profiling in peripheral blood showed significant increases in AEA, OEA, and PEA, which could be further exaggerated in the brain and DRG. Further work is needed to understand which FAA is the major contributor to the painless phenotype.

The microdeletion removes the promoter and first two exons of *FAAH-OUT*, but how this disrupts the function of *FAAH* is still to be elucidated. A hypothesis is that the *FAAH-OUT* transcript normally functions as a decoy for microRNAs as a result of the high sequence homology, and protects *FAAH* mRNA from degradation (Supplementary Fig. S7).^26^ Alternatively, *FAAH-OUT* may have an epigenetic role in regulating *FAAH* transcription, or the deletion removes a critical transcriptional regulatory element.^25^, ^27^ Future work will help us to understand whether targeting *FAAH-OUT* by viral shRNA or gene editing techniques is an effective analgesic/anxiolytic drug development strategy.

This patient provides new insights into the role of the endocannabinoid system in analgesia and more specifically on the *FAAH* genomic locus, and highlights the importance of the adjacent, previously uncharacterised *FAAH-OUT* gene to pain sensation. Given the previous failure of FAAH-inhibitor analgesic drug trials, this report has significance, as it provides a new route to developing FAAH-related analgesia through targeting of *FAAH-OUT*.

## Authors' contributions

Clinical work: HH, JDR, DLHB, DS.

Molecular genetics: AMH, ALO, MCL, SL, SJG, JJC.

Exome sequencing data analyses: JTB.

Bioinformatics: AMH, ALO, JTB, MCL, JJC.

Blood preparation and anandamide analyses: MNH, MvD, MM.

Research design: DS, JJC.

Wrote the manuscript with help from all authors: JJC.

Approved the final manuscript: all authors.

### Supplementary material

Supplementary material is available at *British Journal of Anaesthesia* online.
