# Supplementary material for: Microdeletion in a FAAH pseudogene identified in a patient with high anandamide concentrations and pain insensitivity
Source: Br J Anaesth. 2019 Mar 28;123(2):e249–53. doi: 10.1016/j.bja.2019.02.019 (PMC6676009; doi:10.1016/j.bja.2019.02.019)
Supplement: Multimedia component 1. [file mmc1.docx]

| **DGV accession number** | **Position (hg19)** | **Genomic size** | **Variant type** | **Sample size (individuals)** |
| --- | --- | --- | --- | --- |
| esv2761743 | [chr1:46339858-47026743](http://genome-euro.ucsc.edu/cgi-bin/hgTracks?hgsid=231037144_zBySmMDYLcCUNjWUVeEYa9GnjHLO&db=hg19&position=chr1%3A46339858-47026743) | 686886 | Gain | 2 in 1109 |
| [nsv829748](http://dgv.tcag.ca/dgv/app/variant?id=nsv829748&ref=hg19) | [chr1:46724508-46876122](http://genome-euro.ucsc.edu/cgi-bin/hgTracks?hgsid=231037144_zBySmMDYLcCUNjWUVeEYa9GnjHLO&db=hg19&position=chr1%3A46724508-46876122) | 151615 | Loss | 1 in 95 |
| [nsv546162](http://dgv.tcag.ca/dgv/app/variant?id=nsv546162&ref=hg19) | [chr1:46807597-46870761](http://genome-euro.ucsc.edu/cgi-bin/hgTracks?hgsid=231037144_zBySmMDYLcCUNjWUVeEYa9GnjHLO&db=hg19&position=chr1%3A46807597-46870761) | 63165 | Loss | 1 in 17421 |
| [esv3585936](http://dgv.tcag.ca/dgv/app/variant?id=esv3585936&ref=hg19) | [chr1:46884238-46892335](http://genome-euro.ucsc.edu/cgi-bin/hgTracks?hgsid=231037144_zBySmMDYLcCUNjWUVeEYa9GnjHLO&db=hg19&position=chr1%3A46884238-46892335) | 8098 | Loss | 1 in 2504 |

**Table S1: Structural variants in DGV (database of genomic variants)**
