# Supplementary figures and images for: Microdeletion in a FAAH pseudogene identified in a patient with high anandamide concentrations and pain insensitivity
Source: Br J Anaesth. 2019 Mar 28;123(2):e249–53. doi: 10.1016/j.bja.2019.02.019 (PMC6676009; doi:10.1016/j.bja.2019.02.019)

Fig. S1

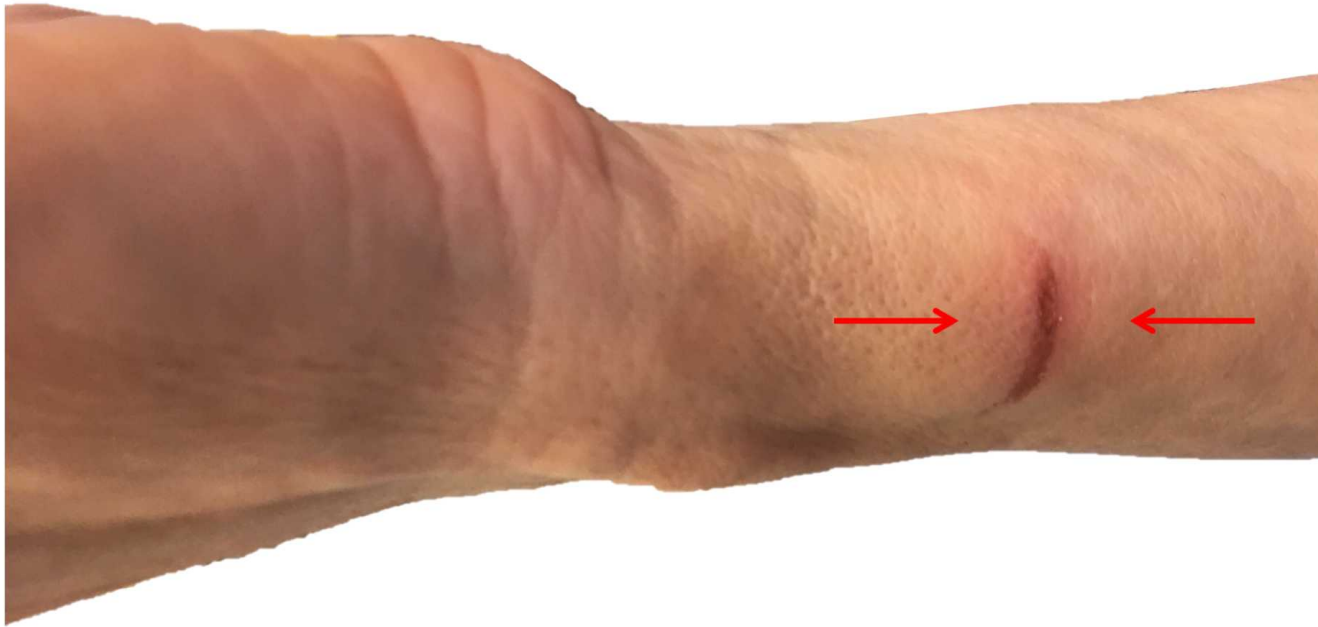

Supplement: Multimedia component 2. [file mmc2.pdf]

Fig. S2

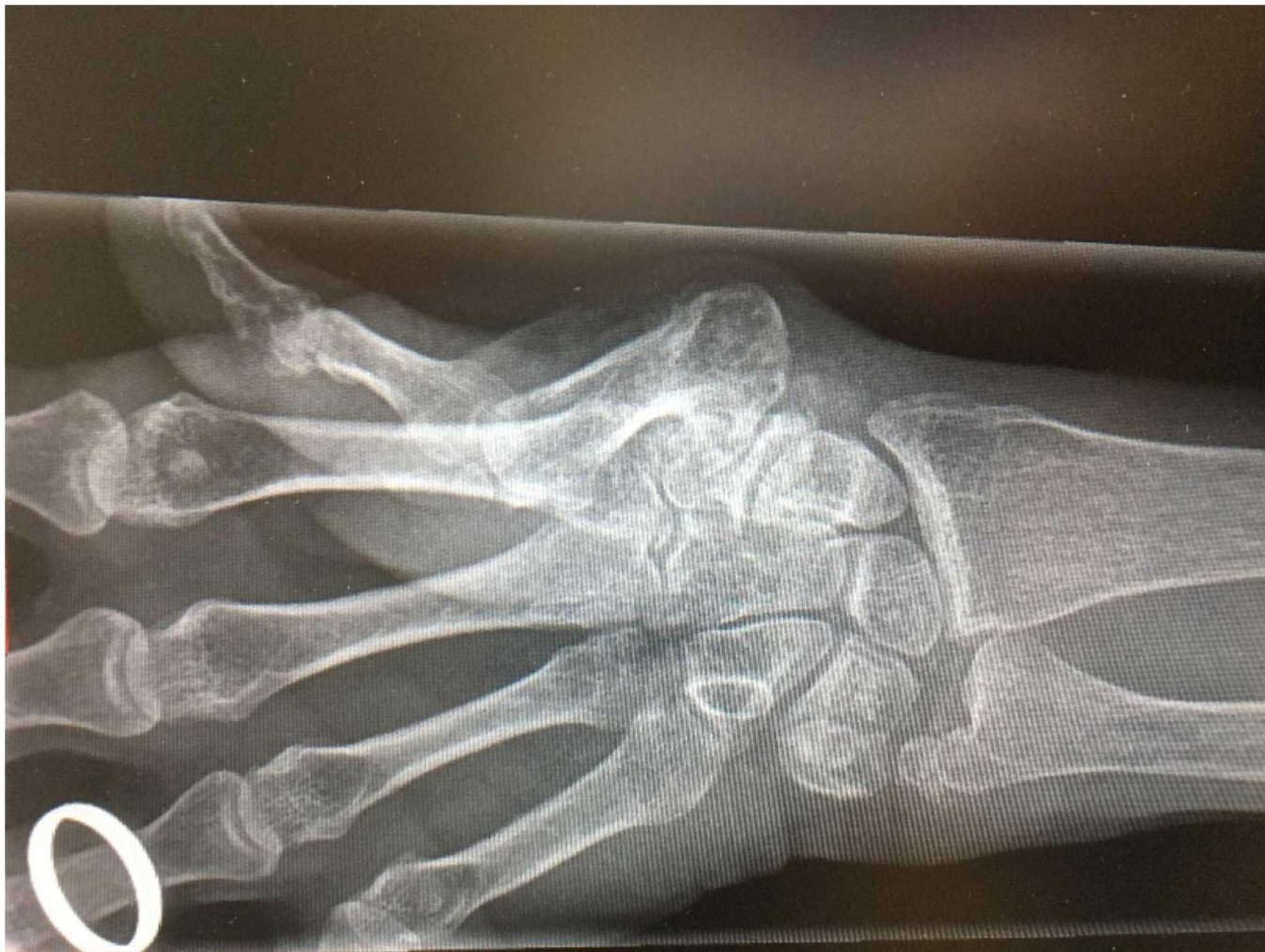

Supplement: Multimedia component 3. [file mmc3.pdf]

Fig. S4

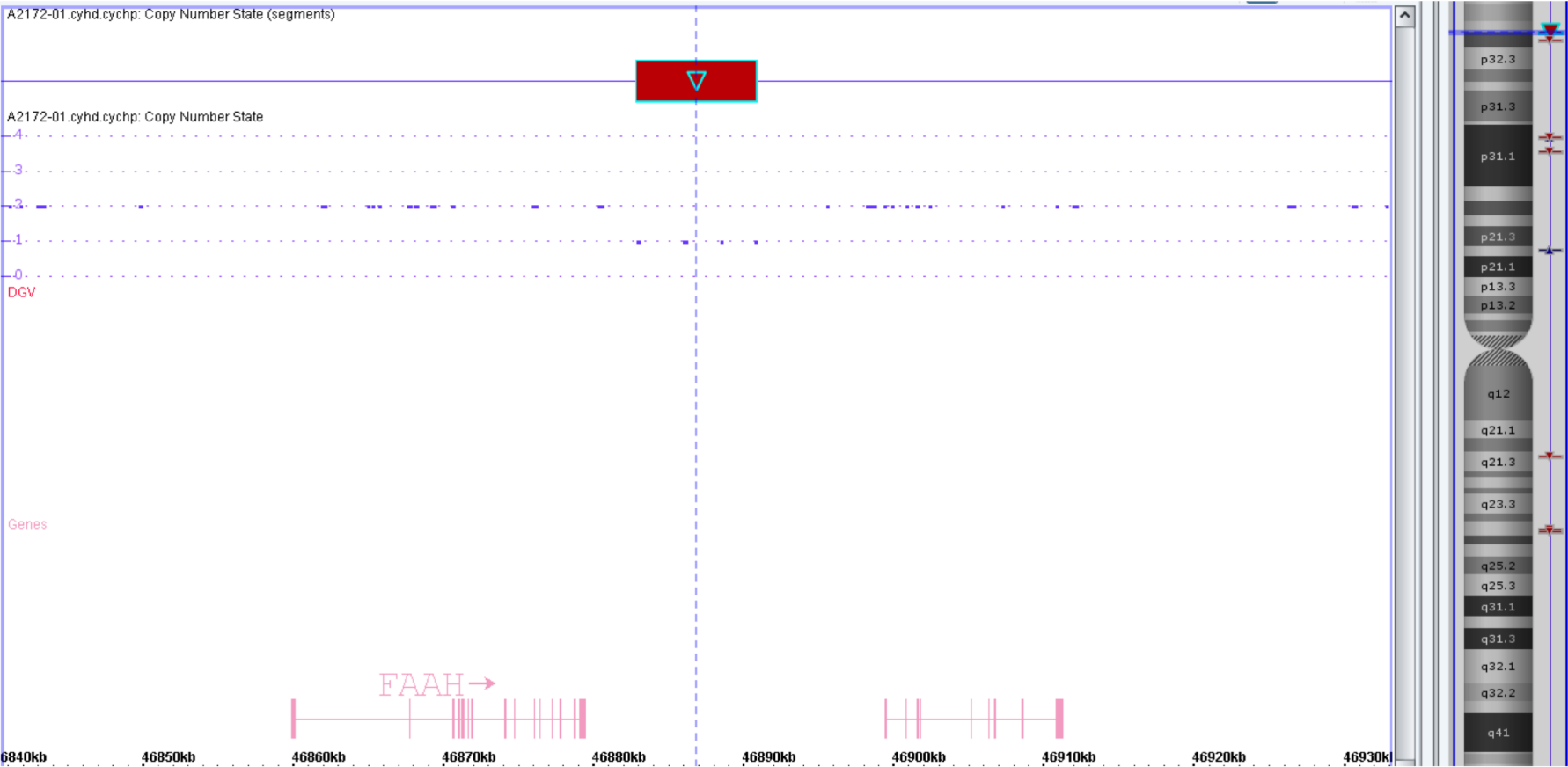

Supplement: Multimedia component 5. [file mmc5.pdf]

Fig. S6

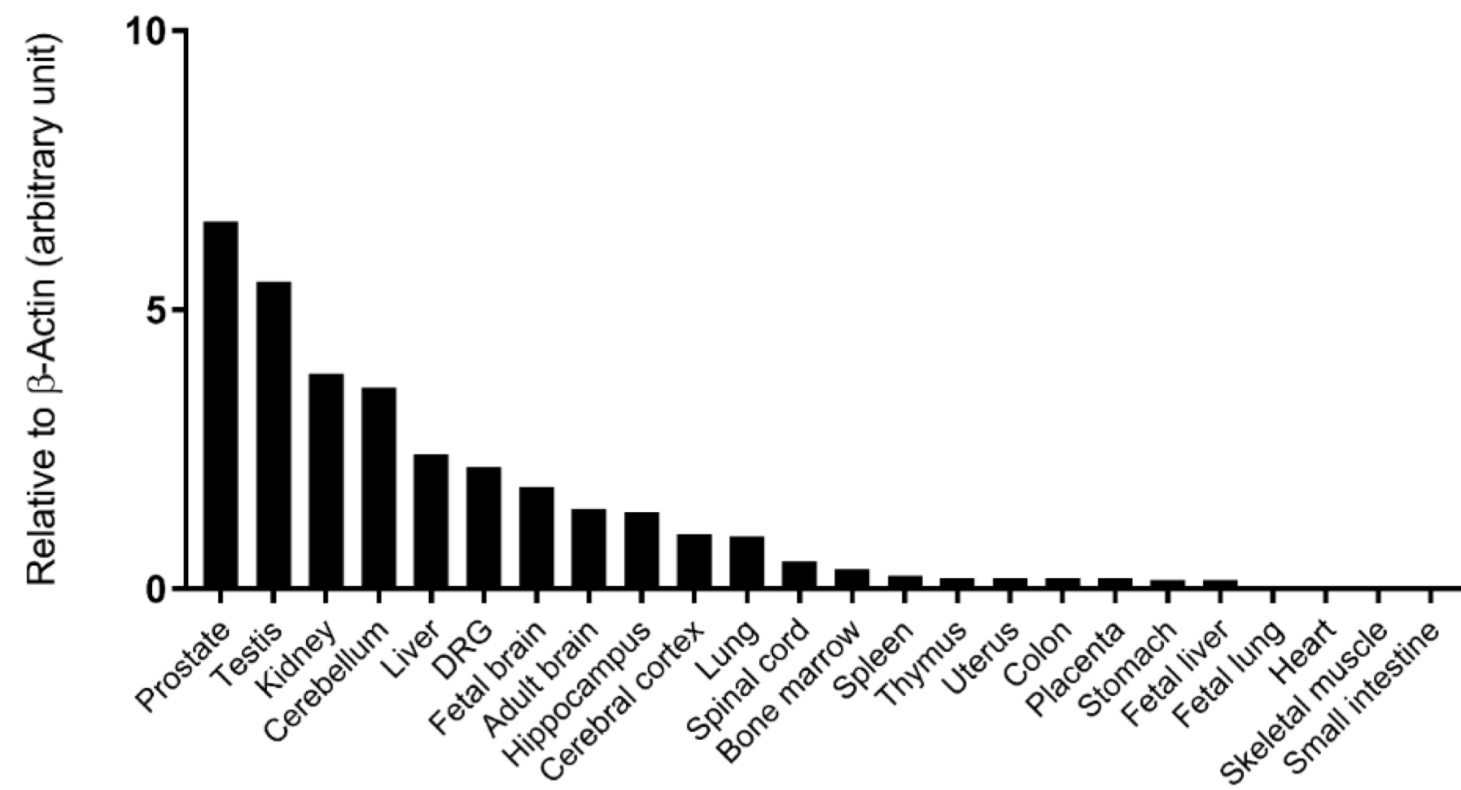

Supplement: Multimedia component 7. [file mmc7.pdf]
