# Supplementary material for: Microdeletion in a FAAH pseudogene identified in a patient with high anandamide concentrations and pain insensitivity
Source: Br J Anaesth. 2019 Mar 28;123(2):e249–53. doi: 10.1016/j.bja.2019.02.019 (PMC6676009; doi:10.1016/j.bja.2019.02.019)
Supplement: Multimedia component 4. [file mmc4.pdf]

Fig. S3

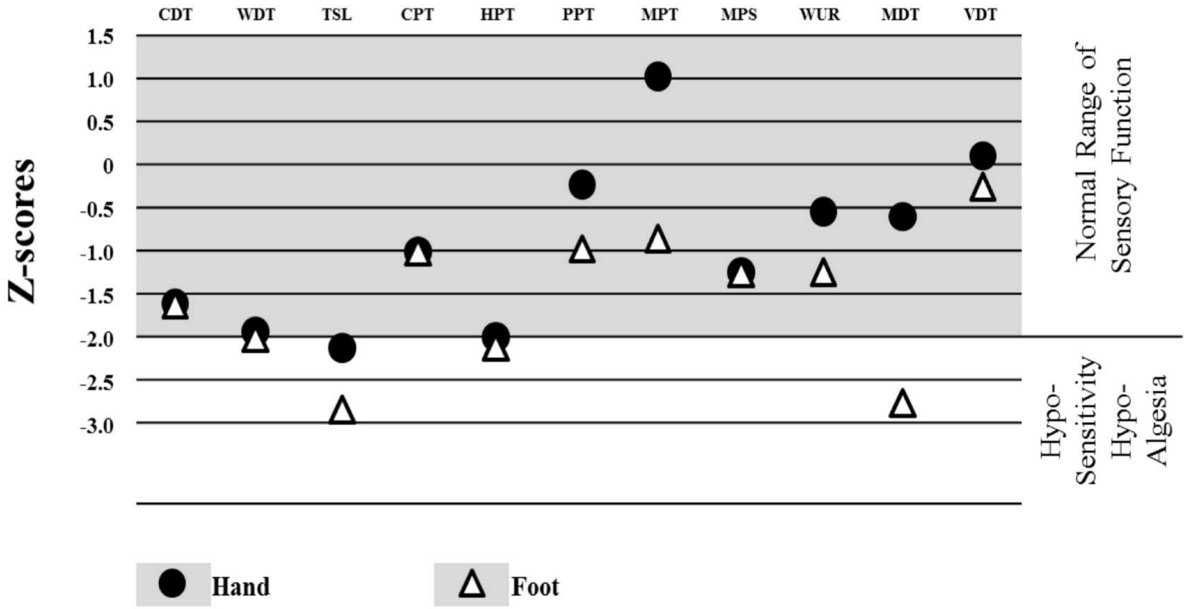

Quantitative Sensory Testing was performed according to the protocol of the German Research Network on Neuropathic Pain. QST is a measurement of sensory perception to a given stimulus. Comparisons of evaluated sites are done against a Caucasian control population for each one of the evaluated sites (in this case hand and foot). Gain of sensory function is presented as a z score >2, and loss of sensory function as <2. Note that in terms of pain modalities there is hyposensitivity in relation to heat pain threshold affecting the hand and the foot. There is also hyposensitivity in non-noxious thermal modalities warm detection threshold and thermal sensory limen. Response to mechanical stimuli is within the normal range other than hyposensitivity to mechanical detection in the foot.

CDT: cold detection thresholds. **WDT:** warm detection thresholds. **TSL:** thermal sensory limen. **CPT:** cold pain thresholds. **HPT:** heat pain thresholds. **PPT:** pressure pain thresholds. **MPT:** mechanical pain thresholds. **MPS:** mechanical pain sensitivity. **WUR:** wind up ratio. **MDT:** mechanical detection thresholds. **VDT:** vibration detection thresholds. **DMA:** dynamic mechanical allodynia. **PHS:** paradoxical heat sensations.
