# Supplementary material for: Microdeletion in a FAAH pseudogene identified in a patient with high anandamide concentrations and pain insensitivity
Source: Br J Anaesth. 2019 Mar 28;123(2):e249–53. doi: 10.1016/j.bja.2019.02.019 (PMC6676009; doi:10.1016/j.bja.2019.02.019)

Fig. S5

A

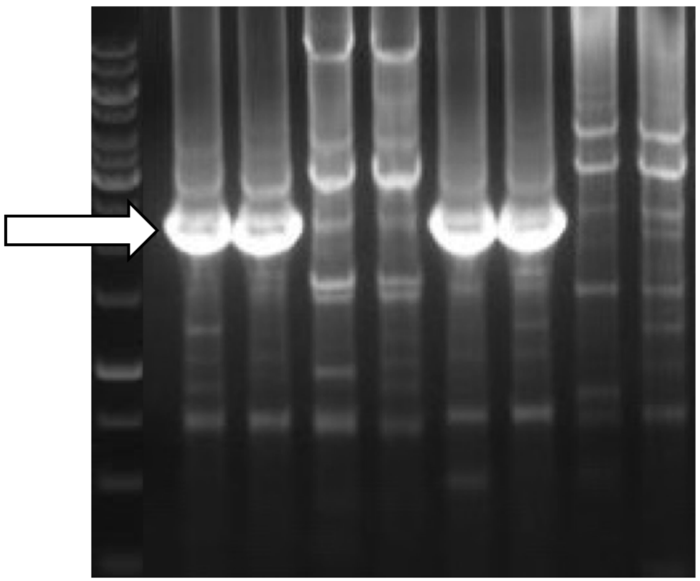

C

```
TTCtttttttttttCGAGACGGAGTTTCGCTCTTGTTGTGCAGGCTGGAGTGCAATGGCG
|| ||||||||||| |||||||||||||||||||||||  ||||||||||||||||||| 
TTTTTTTTTTTTTTGGAGACGGAGTTTCGCTCTTGTTGCCCAGGCTGGAGTGCAATGGCA

AGATCTCAGCTCACTGCAACCTCTGCCTACCGGATTCAAGTGATTCTCCTGCCTCAGCCT
 |||||| |||||| |||||||| |||| || ||||||||  ||||||||  ||||||||
TGATCTCGGCTCACCGCAACCTCCGCCTCCCAGATTCAAGCAATTCTCCTATCTCAGCCT

CCCGAGTATCTGGGATTACAGGCGTGTGCCACCATGCCCAGCTAATTTTGTATTTTTAGT
||| |||| |||||||||||||| ||  |||||| |||||||||||||||||||||||||
CCCAAGTAGCTGGGATTACAGGCATGCACCACCACGCCCAGCTAATTTTGTATTTTTAGT

AGAGATGGGATTTCTCCATGTTAGTCAGGCTGGTCTTGAACTCCTGACCTCAGGTGATCT
||||||||| |||||||||||| |  |||||||||| |||||||||||||||||||||||
AGAGATGGGGTTTCTCCATGTT-G--AGGCTGGTCTCGAACTCCTGACCTCAGGTGATCT

GCCTGCCTTGGCCTCCCAAAGTGCTGGGATTACAGGCGTGAGCCACTGTGCCCGGCCT
|||  ||| |||||||||||||||||||||||||||||| || ||| | |||||||||
GCCCACCTCGGCCTCCCAAAGTGCTGGGATTACAGGCGTAAGTCACCGCGCCCGGCCT
```

B

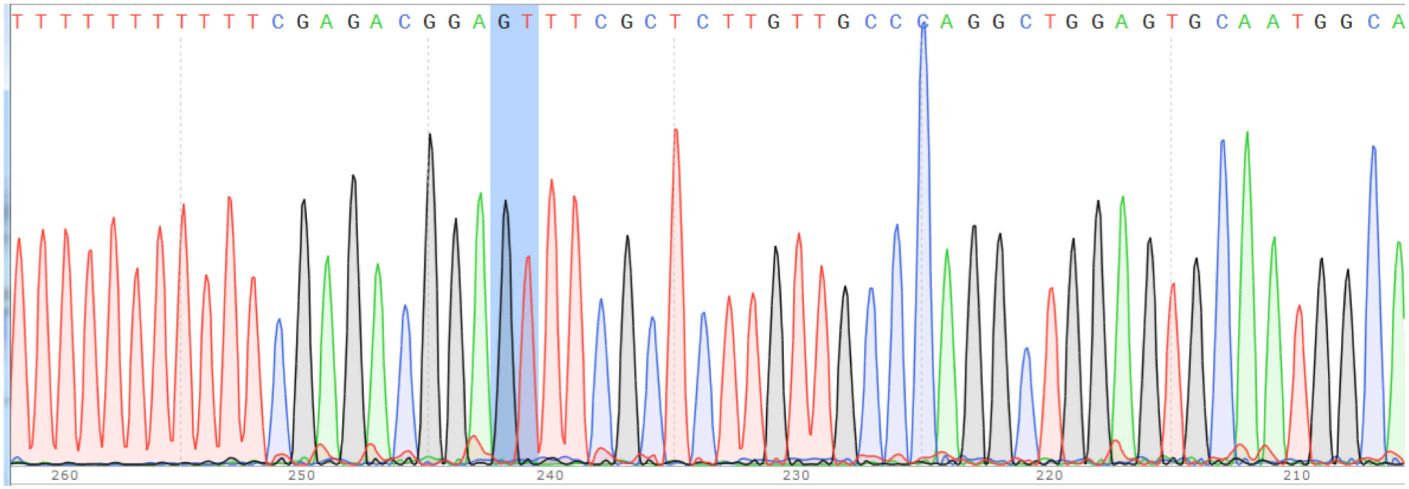

Supplement: Multimedia component 6. [file mmc6.pdf]
