# Supplementary material for: Microdeletion in a FAAH pseudogene identified in a patient with high anandamide concentrations and pain insensitivity
Source: Br J Anaesth. 2019 Mar 28;123(2):e249–53. doi: 10.1016/j.bja.2019.02.019 (PMC6676009; doi:10.1016/j.bja.2019.02.019)
Supplement: Multimedia component 9. [file mmc9.pdf]

Fig. S7

B

```
MLSPVCFLPLLDTSCFGFLAIWSHSLTPKKLWEQHTAVEEYEQEFIAKWR
SLDLDVLLVPVLGSAFYIGSSSLASESQSYVTLYNLLDFPAGVVPVTIVT
LQDEEELAFYKGCYGDSSDKNFSEAVRGSVGLLVTVQCIALPWEEELCLR
FMKEVDTLVKNQRGPK
```

C

69% homology

```
FAAH     438  SRSAGKLWELQHEIEVYRKTVIAQWRALDLDVVLTPMLAPALDLNAPGRATGAVSYTMLY
              S +  KLWE    +E Y +  IA+WR+LDLDV+L P+L  A  + +   A+ + SY  LY
FAAH-OUT 25   SLTPKKLWEQHTAVEEYEQEFIAKWRSLDLDVLLVPVLGSAFYIGSSSLASESQSYVTLY

FAAH     498  NCLDFPAGVVPVTTVTAEDEAQMEHYRGYFGDIWDKMLQKGMKKSVGLPVAVQCVALPWQ
              N LDFPAGVVPVT VT +DE ++  Y+G +GD  DK   + ++ SVGL V VQC+ALPW+
FAAH-OUT 85   NLLDFPAGVVPVTIVTLQDEEELAFYKGCYGDSSDKNFSEAVRGSVGLLVTVQCIALPWE

FAAH     558  EELCLRFMREVERLMTPEK
              EELCLRFM+EV+ L+  ++
FAAH-OUT 145  EELCLRFMKEVDTLVKNQR
```
