# Supplementary material for: Microdeletion in a FAAH pseudogene identified in a patient with high anandamide concentrations and pain insensitivity
Source: Br J Anaesth. 2019 Mar 28;123(2):e249–53. doi: 10.1016/j.bja.2019.02.019 (PMC6676009; doi:10.1016/j.bja.2019.02.019)
Supplement: Multimedia component 10. [file mmc10.docx]

**Supplementary figure legends**

**Fig. S1. Photograph showing burn on right forearm, which was painless**

Burn indicated by arrows. The background of the image has been removed.

**Fig. S2. Radiograph showing severe hand arthritis and deformity prior to hand surgery**

The patient did not feel pain with this degree of arthritis.

**Fig. S3. Quantitative sensory testing**

***Control Site*:** hand, ***Test site*:** foot. The test revealed pathological hyposensitivity in the warm detection thresholds and thermal sensory limen for both hand and foot and some hyposensitivity in the foot for thermal pain thresholds. Mechanical detection thresholds were abnormal in the foot. There was no evidence of paradoxical heat sensations and/or dynamic mechanical allodynia.

**Fig. S4. Identification of microdeletion using the Cytoscan HD array**

Chromosome Analysis Suite (Affymetrix) screenshot showing the heterozygous ~ 8 kb microdeletion identified on chromosome 1 in the patient. Eight consecutive probes (denoted by blue spots) showed a -1 allele copy number and span chromosome 1p33:46,882,936-46,890,857 (build hg19). At the time of analysis, this microdeletion (red box) was not annotated in the Database of Genomic Variants (see empty track in DGV) in figure. A similar deletion has subsequently been reported in 1 out of 5008 alleles in phase 3 of the 1000 Genomes Project (this individual, HG10353, is homozygous WT for *FAAH* SNP rs324420). The microdeletion is located ~4.7kb downstream of the *FAAH* 3’UTR. Non-annotated exons are shown next to the *FAAH* gene at the bottom of the figure. We subsequently extended this novel gene footprint into the microdeletion region using 5’RACE.

**Fig. S5. Validating the microdeletion**

(A) PCR primers located outside of the microdeleted region amplified a 2,259 bp region from the patient and her son (i.e. the allele containing the microdeletion), as indicated by the white arrow. The wild-type allele product from the same reaction is 10,390 bp (beyond the capability of the DNA polymerase). A 1 kb ladder is in lane 1. DNA samples were run in duplicate.

(B) Sanger sequencing of the mutant allele identified the microdeletion breakpoint (the 8,131 bp deleted sequence lies between the highlighted bases).

(C) AluSp sequences (hg38/human; chr1:46418719-46419015 and chr1:46426839-46427143) flank the microdeleted region. Alignment of these sequences shows 89% identity over 298 bp, likely predisposing the region to a genomic rearrangement.

**Fig. S6.** **Real-time qPCR showing expression profile of *FAAH-OUT* in human tissues**

*FAAH-OUT* is expressed in a wide range of human tissues, notably in dorsal root ganglia and several brain regions. Expression is shown relative to beta actin.

**Fig. S7. Comparison of *FAAH* and *FAAH-OUT* cDNA sequences**

(A) Alignment between *FAAH* and *FAAH-OUT* exonic sequences identify an 800 bp region with ~70% nucleotide identity. The high sequence homology means that the genes potentially share microRNA seed sites, with two examples shown in red text (miR-125a-5p/125b-5p/351/670/4319) and orange text (miR-128/128ab), as predicted by miRcode.

(B) Most likely peptide generated from *FAAH-OUT* cDNA, as predicted by ATG^pr^.

(C) The putative FAAH-OUT protein shares 69% homology (50% identity) over a 139 amino acid segment of the FAAH enzyme.

**Table S1. Structural variants in DGV (database of genomic variants)**

Structural variants curated within the database of genomic variants that map to the *FAAH* or *FAAH-OUT* genomic region.
