# Supplementary material for: Microdeletion in a FAAH pseudogene identified in a patient with high anandamide concentrations and pain insensitivity
Source: Br J Anaesth. 2019 Mar 28;123(2):e249–53. doi: 10.1016/j.bja.2019.02.019 (PMC6676009; doi:10.1016/j.bja.2019.02.019)
Supplement: Multimedia component 11. [file mmc11.docx]

**Supplementary data**

**Supplementary material and methods**

**Additional clinical information**

PFS (the proband) was born in 1947 and reported no problems with walking or speech, and other developmental milestones were achieved without issues. Education was unremarkable and she completed primary school teacher training and has a diploma in special needs education. She has no problems in smelling and can tell the difference between coffee, orange, perfume, mint and she enjoys spices and different flavours. Mosquito bites cause her inflammation (redness and swelling) and nettle stings are noted as pleasant and not painful. PFS has never smoked tobacco and very occasionally consumes alcohol. She has been a vegetarian for 38 years and a vegan for 11 years and there is no clinical history of vitamin deficiencies. Of note, her dental surgeon observed, most unusually, that her saliva dissolves the fixative for a temporary denture after just 90 mins.

Menstruation began at 11 years-of-age with periods being uncomfortable, causing her bloating, and a general feeling of unpleasantness. She did not complain about pain during her two childbirths at 29 and 42 years of age (although remembers receiving gas analgesia). She recalls labour as causing her a sensation of pressure, felt unpleasant, but not painful. She required stitches for a tear but no painkillers were required.

Upon examination at 67 years-of-age her height was 170 cm and weight was 63 kg. Blood pressure was 105/75 mmHg lying with no postural drop. Examination of the cranial nerves was normal and there was no muscle weakness or significant motor abnormalities in the limbs. All deep tendon reflexes were preserved. In the upper limbs light touch, joint position sense and vibration were preserved distally, but temperature sensation was lost to the wrists bilaterally and pin prick sensation was impaired to the shoulder (i.e. it was felt as a touch but did not elicit the sharp pricking painful sensation). In the lower limbs touch, joint position sense and vibration were also preserved distally. Temperature sensation was lost to the base of the toes and pinprick sensation was lost to the knees. The intra epidermal nerve fibre density from the distal leg (10 cm above the lateral malleolus) was 5.8 f/mm and within normal range for age and gender.^1^ Nerve conduction tests demonstrated normal motor and sensory conduction parameters. She refers to a burning sensation in her toes due to the hallux valgus which is accompanied by tingling and pins and needles which she describes as being “pleasant”.

Her son also reports of having some degree of pain insensitivity but not to the same extent as his mother. He does not feel pain from donating blood or from cuts or bruises and frequently scalds his mouth with hot drinks and food, not realising until skin starts to peel off. He reports never having the need to take pain killers.

**Quantitative Sensory Testing (QST)**

QST was performed following informed consent from the proband on the dorsum of the foot and the hand according to the German neuropathic pain network (DFNS) protocol by a DFNS trained experimenter.^2^ This protocol includes 13 parameters which are designed to detect both gain and loss of function and is summarised below:

Thermal stimuli: Temperature testing was conducted using a TSA-2001-II (Medoc, Israel) with a thermode of contact area 9 cm². Thresholds were obtained using ramped stimuli (1°C/s) from 32°C (centre of neutral range) until terminated by subject-control or automated cut-off temperatures whereby the temperature would return to baseline temperature. Thermode temperature was held at 32°C during 10 s inter-stimulus intervals. Sequentially, cooling and warming detection thresholds (CDT, WDT) were assessed. The presence of paradoxical heat sensations (PHS) was investigated by alternating warming and cooling which were also used to determine thermal sensory limen (TSL). Cold and heat pain thresholds (CPT, HPT) were then measured. Tests were performed in triplicate and mean data used for comparison. The subject was unaware of the timing of initiation of temperature increase and inter-stimuli intervals.

Mechanical stimuli: Mechanical detection threshold (MDT) was assessed using a standardized set of identically round-tipped von Frey hairs (Optihair_2_-Set, Marstock, Nervtest, Germany) of two-fold incremental bending forces within the range 0.25 – 512 mN. MDT was calculated as a geometric mean of five thresholds ascertained using sequential ascending and descending applications of the hairs by a ‘method of limits’. Assessment of vibration sense (VS) was performed with a Rydel–Seiffer graded tuning fork (64 Hz, 8/8 scale) that was placed over the processus styloideus ulnae or malleolus internus and left there until the subject could not feel vibration any more and was performed three times. Mechanical pain threshold (MPT) were ascertained using a set of seven mechanical probes which exert fixed intensities of 8, 16, 32, 64, 128, 256, 512 mN with a blunt contact area of 0.2 mm diameter. Stimuli were applied in ascending sequence at a rate of 2 s on, 2 s off until a stimulus was perceived as sharp and subsequent descending until no longer reported as such. Threshold was calculated as the geometric mean of five of these series. Wind up ratio (WUR) is a perceptual model of temporal spinal wind up was generated from the perceived intensity of painfulness from a single application of 256 mN pinprick compared to 10 repetitions of the same stimulus applied at a rate of 1/second within a 1 cm^2^ area. Pain intensity was reported using a numerical rating scale (NRS) where 0 represents no pain and 100, maximal imaginable pain. The process was repeated five times and WUR calculated by dividing the mean pain report following the ten applications, by the mean intensity reported from single stimuli. Mechanical pain sensitivity (MPS) was assessed using the same set of seven weighted pinprick stimuli to obtain a stimulus–response function for pinprick-evoked pain (the strongest pinprick force was about eight times the mean mechanical pain threshold). The subject was asked to give a pain rating for each stimulus on a ‘0–100’ numerical rating scale (‘0’ indicating “no pain”, and ‘100’ indicating “most intense pain imaginable”). This test was designed to detect pinprick hyperalgesia. Dynamic mechanical allodynia (DMA) was assessed as part of this test using a cotton wisp, a cotton wool tip and a standardised brush (Somedic Sweden) exerting a force of 200-400 mN. Pressure pain threshold (PPT) was assessed at the thenar eminence and instep using a pressure gauge devise (FDN 200, Wagner instruments, USA) with a probe area of 1 cm^2^. Pressure pain threshold was determined using three series of ascending stimulus intensities at an increasing ramp of 50 KPa/s.

The findings for each parameter are compared with a control Caucasian population by means of *Z*-scores. The *Z*-score represents the result of a raw score minus the mean of the population and this is further divided by the standard deviation of the population. *Z* scores above or below ±2 standard deviations represent hyper-/hypo-sensitivity and hyper-/hypo-algesia. In the cases when upper limits allowed by the ethics committee for pain testing were reached, the cut-off value expressed is equivalent to the maximum stimulation before it causes any tissue damage (0° to 52°C for temperature, 512mN for mechanical pain, 10Kg for pressure pain).

**Skin biopsy**

A punch skin biopsy was taken from 10 cm above the lateral malleolus of the leg of the proband and fixed overnight with 2% periodate-lysine-paraformaldehyde and preserved in sucrose before blocked and processed into 50 μM sections. Nerve fibres were stained using rabbit anti-PGP (protein gene product) 9.5 Ab (1:2000; Ultraclone Ltd, Yarmouth, Isle of Wight, UK) and Cy3 anti-rabbit (1:500; Jackson Immunoresearch, West Grove, PA, USA). By means of a Zeiss LSM 710 confocal microscope, z-stacks (2 μm intervals), maximum intensity projections were generated with a Plan-Apochromat objective at 20× magnification (Carl Zeiss MicroImaging GmbH, Jena, Germany). Analysis was performed as per published guidelines.^3^ PGP 9.5-positive nerve fibres crossing the dermal-epidermal junction were counted and IENFD counts are given in number of fibres per millimetres of skin.

**Whole exome sequencing**

Exome sequencing was performed to identify a pathogenic mutation for this novel disorder. Given the partial phenotype in the son we predicted a dominant inheritance pattern with variable expressivity. We also considered that PFS may be presenting with a full phenotype due to the inheritance of an additional loss of function allele. Given the previously undescribed phenotype, we searched for novel variants in PFS’s exome which were absent in her unaffected mother and daughter, but inherited by her son.

For enrichment of exons and flanking intronic sequences we used the Agilent Human SureSelect V5 kit with UTRs. We performed 100 bp paired-end runs on a Genome Analyzer HiSeq 2000 system (Illumina) generating sequences of 5.2 (PFS), 5.3 (son), 5.4 (mother) and 5.6 (daughter) Gb. This amount of data resulted in the following percentages of targets being covered at greater than or equal to 10x: 95.7 (PFS), 96 (son), 96 (mother) and 96.2 (daughter). Sequence alignment and variant calling was performed against the reference human genome assembly (hg19) by using the Burrows-Wheeler Aligner^4^ and the Genome Analysis Toolkit.^5,6^ Format conversion and indexing were performed with the Picard software. Single nucleotide variants and small insertions and deletions were checked against established databases (1000 Genomes Project and dbSNP v.142). Variants were further checked using the ExAC browser, dbSNP v.150 and in our in-house database of sequencing data for other diseases (n>2000). The protein coding effects of variants was predicted using SIFT, Polyphen2 and M-CAP. Splicing changes were analysed using the NNSPLICE Splice Site Predictor. Novel variants were verified by Sanger sequencing and checked to see how they segregated within the family (primers available on request).

Following filtering of variants we identified 4 candidate mutations in PFS and her son, but none of which we considered as likely to be causal for the phenotype: *MACF1* (NM_012090:c.C14416T:p.L4806F); *USP24* (NM_015306:c.G6490A:p.V2164M); *KIAA1107* (NM_015237:c.A3359G:p.N1120S) and *NSD1* (NM_022455:c.C6703T:p.H2235Y). The variants in *USP24*, *KIAA1107* and *NSD1* are annotated as benign by the Polyphen2 (HumVar) tool. The *MACF1* variant is annotated as ‘probably damaging’ and is a neural gene. However, we considered this microtubule-actin crosslinking factor to be a low priority candidate following an analysis of known gene functions in relation to pain.

**Genomic copy number analyses**

Genomic DNA isolated from a peripheral blood sample from PFS was used for the Cytoscan HD Copy Array (Affymetrix) and run by AROS according to the manufacturer’s conditions. Data was analysed using Chromosome Analysis Suite (Affymetrix) software and novel genomic variants identified by comparison to the Database of Genomic Variants (DGV).

**Deletion breakpoint cloning**

A range of primers were designed that were predicted to flank the microdeletion identified in the Cytoscan HD Copy Array. A 2,259 bp product was amplified from PFS and the son using LA Taq DNA Polymerase (Clontech) and the primers 5’CCACCAGTGTGCTGGTGGCTAC and 5’AGCCTCTGGGGCACTTTGACTC (Fig. S5A). Primers closer to the deletion breakpoints were then designed and used to amplify a 1349 bp product using KAPA HiFi DNA Polymerase and the primers 5’TTAATGTCTGGAGTGATAACATGAC and 5’ACAACTTCTAATTAGTGTTAATGAC. Sanger sequencing of the gel purified PCR product (Qiagen) using primers 5’ TTAATGTCTGGAGTGATAACATGAC and 5’ AAGGCCGGGCGCGGTGACTTAC enabled identification of the microdeletion breakpoints (Fig. S5B).

**PCR amplification and Sanger sequencing of SNP rs324420**

Genomic DNA was used as template to amplify a 424 bp product from the region containing SNP rs324420 using primers 5’ CTCTGGGCCATGTTGCTGGTTAC and 5’ CAACTGTCACACAGGCCAAAACAG. Purified PCR products were Sanger sequenced by standard methods.

**Cloning *FAAH-OUT***

Partial expressed sequence tags (ESTs) were identified downstream of the microdeletion that in Refseq were assembled into a 1267 bp sequence (NR_045483) and annotated as ‘fatty acid amide hydrolase pseudogene (*FAAHP1*) non-coding RNA’. To identify further 5’exons, the *FAAHP1* locus from a variety of species was searched using the UCSC genome browser. This led to the identification of EST CN788775 in the cow genome. The nucleotide sequence of this EST was compared to the human genome using the Blat tool and consensus splice donor and acceptor sites identified within the human genomic DNA sequence. Human adult brain total RNA was reverse transcribed into cDNA using oligo d(T) and the Superscript III first-strand synthesis system (Invitrogen). A forward primer mapping to the most 5’ predicted exon (5’ CCAGAAGTGGAGGGAGGTAGCAC) and a reverse primer in a downstream predicted exon (5’ GCTGTCATAGGTGTCCTTGAGGCTC) were designed and used to amplify a product from human adult brain cDNA. Sanger sequencing confirmed the amplicon to be novel human exons mapping 5’ to *FAAHP1*. Next, 5’RACE was carried out using whole human brain Marathon-Ready cDNA (Clontech) using reverse primer (5’ CAAAGTGAGACTCCGTCTGCTGC) according to the manufacturer’s conditions. The generated amplicon was cloned into the pCR-Blunt II-TOPO (Thermofisher) and sequenced using M13 forward and reverse primers. A forward primer (5’ ggcaaaggcgccattctcctgggtaca) was then designed at the most 5’ end of the newly identified transcript and a reverse primer (5’gccagtcagaaaatgtttattgagctc) in the most 3’ exon of NR_045483. Human cerebral cortex cDNA was used as template to amplify a 2845 bp product, which was subsequently cloned into pCR-Blunt II-TOPO and fully sequenced. The insert of the sequenced clone (CC2) has been submitted to GenBank under accession number KU950306 and we name the gene *FAAH-OUT*.

**Real-time qPCR analysis in human tissues**

One μg of total RNA derived from a range of human tissues (Clontech) was reverse transcribed using oligo d(T) and Superscript III first-strand synthesis system (Invitrogen) according to the manufacturer’s conditions. Real time PCR was carried out using the Universal SYBR Green Supermix protocol (Bio-Rad) and the following primers: *FAAH-OUT*, 214 bp (5’ ACTGACACAGGTGACAGCATCTG and 5’ GTCCAGTCGGTACATGTCTTCAC); and Actin (*ACTB*), 144 bp (5’ CCTGGCACCCAGCACAAT and 5’ GGGCCGGACTCGTCATACT). These assays were performed on the BioRad CXF Connect^TM^ real-time thermal cycler. *FAAH-OUT* expression was compared with that of Actin measured on the same sample in parallel on the same plate, giving a CT difference (ΔCT) for *ACTB* minus the test gene. Mean and standard error were performed on the ΔCT data and converted to relative expression levels (2ˆΔCT).

**Assessment of plasma endocannabinoids (EC)**

Levels of AEA, PEA, OEA and 2-AG were measured by mass spectrometry from blood samples taken from PFS and 4 unrelated normal controls. Study participants were PFS (index patient, female, post-menopausal (aged 69), *FAAH-OUT* microdeletion and A/C for rs324420); control A (female, post-menopausal (aged 50), CC for rs324420); control B (male, aged 57, CC for rs324420); control C (female, post-menopausal (aged 50), AC for rs324420) and control D (male, aged 49, AC for rs324420). Study participants were free of all medications for at least four weeks. Whole blood, anticoagulated with EDTA, was obtained between 8-9 AM following overnight fasting through venipuncture of a forearm vein. Ex vivo blood was settled on ice for <20 mins and centrifuged at 4^o^C and plasma stored at -80^o^C until assayed. Assay of plasma levels of endocannabinoids N-arachidonoylethanolamide (AEA) and 2-arachidonoylglycerol (2-AG) was performed using a previously published method.^7^ In brief, 500 μl of plasma was directly pipetted into 2ml of acetonitrile, to which the internal standards [^2^H_8_]-AEA (5 pmol) and [^2^H_8_]-2-AG (5 nmol) (Cayman Chemicals, Ann Arbor, MI) had already been added. All samples were sonicated and stored at -20^o^C overnight to precipitate proteins. The following morning all samples were centrifuged at 1500 x g for 4 minutes, after which the supernatant was transferred to a new vial and centrifuged again under the same parameters. The supernatant from this second centrifugation was then transferred to a clean borosilicate glass tube and dried down under nitrogen gas. All samples were then resuspended in 200ul of acetonitrile and stored at -80^o^C until analysis. AEA and 2-AG were quantified using liquid chromatography and tandem mass spectrometry (LC-MS/MS) as described previously^7^ and all values were normalized to concentration per ml plasma.

**Supplementary references**

1. Bakkers, M. *et al.* Intraepidermal nerve fiber density and its application in sarcoidosis. *Neurology* **73**, 1142-8 (2009).

2. Rolke, R. *et al.* Quantitative sensory testing in the German Research Network on Neuropathic Pain (DFNS): standardized protocol and reference values. *Pain* **123**, 231-43 (2006).

3. Lauria, G. *et al.* European Federation of Neurological Societies/Peripheral Nerve Society Guideline on the use of skin biopsy in the diagnosis of small fiber neuropathy. Report of a joint task force of the European Federation of Neurological Societies and the Peripheral Nerve Society. *Eur J Neurol* **17**, 903-12, e44-9 (2010).

4. Li, H. & Durbin, R. Fast and accurate short read alignment with Burrows-Wheeler transform. *Bioinformatics* **25**, 1754-60 (2009).

5. DePristo, M.A. *et al.* A framework for variation discovery and genotyping using next-generation DNA sequencing data. *Nat Genet* **43**, 491-8 (2011).

6. McKenna, A. *et al.* The Genome Analysis Toolkit: a MapReduce framework for analyzing next-generation DNA sequencing data. *Genome Res* **20**, 1297-303 (2010).

7. Qi, M., Morena, M., Vecchiarelli, H.A., Hill, M.N. & Schriemer, D.C. A robust capillary liquid chromatography/tandem mass spectrometry method for quantitation of neuromodulatory endocannabinoids. *Rapid Commun Mass Spectrom* **29**, 1889-97 (2015).
