# Supplementary material for: Microdeletion in a FAAH pseudogene identified in a patient with high anandamide concentrations and pain insensitivity
Source: Br J Anaesth. 2019 Mar 28;123(2):e249–53. doi: 10.1016/j.bja.2019.02.019 (PMC6676009; doi:10.1016/j.bja.2019.02.019)
Supplement: Multimedia component 8. [file mmc8.pdf]

A

Fig. S7

68% identity

```
FAAH      476  GGCCTGCTCTATGGCGTCCCTGTGAGCCTCAAGGAGTGCTTCACCTACAAGGGCCAGGAC
               ||||| |||||||| |||||  |||||||||||||   ||    |  || |||||| |||
FAAH-OUT 1363  GGCCTTCTCTATGGGGTCCCCATGAGCCTCAAGGACACCTATGACAGCATGGGCCATGAC

FAAH      536  TC--CACGC----TGGGCTTGAGCCTGAATGAAGGGGTGCCGGCGGAGTGCGACAGCGTA
               |   || ||    ||| || |  |||| | ||||   ||| | |  ||   ||| | ||
FAAH-OUT 1423  TGTGCATGCCGCCTGGCCTAGTTCCTGGA-GAAGCC-TGC-GACCAAG---GACGGGGTC

FAAH      590  GTGGTGCATGTGCTGAAGCTGCAGGGTGCCGTGCCCTTCGTGCACACCAATGTTCCACAG
                |  || | ||||| |||   || || ||| | ||||| ||  | ||||| |  |||| |
FAAH-OUT 1477  ATTATGAAAGTGCTCAAGGCCCAAGGAGCCATCCCCTTTGTTAAGACCAACATCCCACTG

FAAH      650  TCCATGTTCAGCTATGACTGCAGTAACCCCC-TCTTTGGCCAGACCGTGAACCCATGGAA
                |  || |||||| ||| ||| | ||| ||| ||| ||||||||   ||| ||||  |||
FAAH-OUT 1537  ACGCTGCTCAGCTTTGAATGCGGCAACTCCCATCTATGGCCAGATGTTGAGCCCACTGAA

FAAH      709  GTCCTCCAAAAGCCCAG--GGGGCTCCTCAGGGGGTGAAGGGGCCCTCATCGGGTCTGGA
                |  |  | ||| |  |  ||||||||||||||||||| ||||| ||  | |      |
FAAH-OUT 1597  CT--TAAAGAAGACATGTGGGGGCTCCTCAGGGGGTGA-GGGGCTCTGCTGGCAGAAAGG

FAAH      767  GGCTCCCCCCTGGGCTTAGGCACTGATATCGGAGGCAGCATCCGCTTCCCCTCCTCCTTC
               || |||  ||| ||| | || ||||| |  || | ||||||| || | ||  ||  ||||
FAAH-OUT 1654  GGGTCCATCCTAGGCATGGGTACTGACACAGGTGACAGCATCTGCATACCAGCCAGCTTC

FAAH      827  TGCGGCATCTGCGGCCTCAAGCCCACAGGGAACCGCCTCAGCAAGAGTGGCCTGAAGGGC
               || ||  | |  ||||||  | |||||||   |||||||||| | | |||  |    | |
FAAH-OUT 1714  TGTGGTGTTTATGGCCTCTGGACCACAGGTTCCCGCCTCAGCTACACTGGAATT---GCC

FAAH      887  TGTG---TCTATGG-ACAGGAGGCAGTGCGTCTCTCCGTGG---GCCCCATGGCCCGGGA
               | ||   || | || | |  |  | |||     | | ||||   |||||||||||| |||
FAAH-OUT 1771  TCTGCCATCAAGGGGAAAAAAATCGGTGAC---CACGGTGGCTGGCCCCATGGCCCAGGA

FAAH      940  CGTGGAGAGCCTGGCACTGTGCCTGCGAGCCCTGCTGTGCGAGGACATGTTCCGCTTGGA
               ||||||||||||||| |||||||||| |||||||||| | || ||||||| |||  ||||
FAAH-OUT 1828  CGTGGAGAGCCTGGCGCTGTGCCTGCAAGCCCTGCTGAGTGAAGACATGTACCGACTGGA

FAAH     1000  CCCCACTGTGCCTCCCTTGCCCTTCAGAGAAGAGGT
               |||||||||||  |   ||||||| || || |||||
FAAH-OUT 1888  CCCCACTGTGCTCCAGATGCCCTTTAGGGAGGAGGT
```

71% identity

```
FAAH     1568  CTGTACAACTGCCTGGACTTCCCTGCAGGGGTGGTGCCTGTCACCACGGTGACTGCTGAG
               |||||||||  ||||||||| || || || |||||||||||||| |  |||||     ||
FAAH-OUT 2216  CTGTACAACCTCCTGGACTTTCCCGCGGGCGTGGTGCCTGTCACTATCGTGACACTACAG

FAAH     1628  GACGAGGCCCAGATGGAACATTACAGGGGCTACTTTGGGGATA--TCTGGGACAAGATGC
               |||||||   |  |||     |||| ||| | ||  || ||||  ||||  | ||  | |
FAAH-OUT 2276  GACGAGGAGGAACTGGCCTTCTACAAGGGGTGCTACGGAGATAGTTCTGACAAAAATTTC

FAAH     1686  TGCAGAAGGGCATGAAGAAGA-GTGTGGGGCTGCCGGTGGCCGTGCAGTGTGTGGCTCTG
               | ||||  |||   |||| ||   || || || | |||| | ||||||||  | ||| ||
FAAH-OUT 2336  T-CAGA--GGCGGTAAGAGGATCCGTCGGACTTCTGGTGACTGTGCAGTGCATTGCTTTG

FAAH     1745  CCCTGGCAAGAAGAGTTGTGTCTGCGGTTCATGCGGGAGGTGGA
               || ||| |||| ||| ||||||| |||||||||  |||||||||
FAAH-OUT 2393  CCATGGGAAGAGGAGCTGTGTCTCCGGTTCATGAAGGAGGTGGA
```
